# Psychological influences and implications for household disaster preparedness: a systematic review

**DOI:** 10.3389/fpubh.2025.1457406

**Published:** 2025-03-14

**Authors:** Minrui Ni, Liang Xia, Xinru Wang, Yixuan Wei, Xiaofei Han, Yiqiao Liu, Song Pan

**Affiliations:** ^1^Department of Architecture and Built Environment, Faculty of Science and Engineering, The University of Nottingham Ningbo China, Ningbo, China; ^2^Center for Sustainable Energy Technologies, University of Nottingham Ningbo China, Ningbo, China; ^3^Research Centre for Fluids and Thermal Engineering, University of Nottingham Ningbo China, Ningbo, China; ^4^College of Mechanical Engineering, Tianjin University of Commerce, Tianjin, China; ^5^Key Laboratory for Comprehensive Energy Saving of Cold Regions Architecture of Ministry of Education, Jilin Jianzhu University, Changchun, China; ^6^School of Civil Engineering and Resources, University of Science & Technology Beijing, Beijing, China; ^7^Centre for Building, Construction and Tropical Architecture, Faculty of Built Environment, Universiti Malaya, Kuala Lumpur, Malaysia; ^8^Department of Mechanical Engineering, Universiti Malaya, Kuala Lumpur, Malaysia; ^9^Beijing Key Laboratory of Green Built Environment and Energy Efficient Technology, Beijing University of Technology, Beijing, China

**Keywords:** psychological factors, household preparedness, psychological interventions, natural disasters, emergency preparedness, systematic review

## Abstract

**Introduction:**

Household disaster preparedness remains critical yet underachieved, despite substantial investments in mitigation infrastructure. Understanding psychological drivers affecting the implementation of household preparedness measures helps distinguish families fully prepared for disasters from those not, thereby improving disaster education. Psychological drivers may promote, hinder, or have no impact on household preparedness. This review fills a significant gap by systematically categorizing psychological factors influencing household disaster preparedness, an area that remains underexplored in previous literature, aiming to provide recommendations for developing more effective psychological interventions and coping mechanisms.

**Methods:**

A systematic literature search was conducted using PRISMA guidelines, analyzing published studies (2017-2024) from Web of Science, Google Scholar, and ScienceDirect. Two authors determined the eligibility of studies based on the inclusion and exclusion criteria.

**Results:**

A total of 35 studies were included in this review. Regarding cognitive appraisal, risk perception generally promotes household preparedness. Hazard intrusiveness, perceived efficacy, and perceived response efficacy encourage preparedness. Regarding motivation and intention, self-efficacy and perceived benefits boost preparedness, and the transfer of responsibility regulates the influence of trust on household preparedness. Regarding social interaction, formal support, and community resilience promotes preparedness, whereas informal support and social norms may impede it. Regarding bonds with the living environment, place attachment promotes housing protection but hinders relocation. Sense of place hinders permanent evacuation or relocation.

**Discussion:**

Disaster prevention and management should emphasize the responsibility of individuals and families in reducing disaster risks, clarify the consequences and probabilities of disasters, refine social norm indicators, and develop a resettlement planning incorporating place identity cultivation to improve effective household preparedness.

## 1 Introduction

Natural disasters are catastrophic events that severely disrupt community or societal functions, leading to significant loss of life, economic damage, and environmental destruction (1–3). Disasters leave an enduring psychological imprint, with documented cases of Post-Traumatic Stress Disorder (PTSD), anxiety, and depression profoundly affecting survivors’ mental health (4, 5). This situation underscores the need for individuals to minimize risk exposure by developing disaster preparedness. As a crucial aspect of disaster risk management, disaster preparedness enhances public understanding and adaptation to risks, aiding in more effective resource utilization for coping (6). Disaster preparedness can be defined as individuals’ knowledge, capabilities, and actions for accurately predicting, responding to, and recovering from disaster impacts (6, 7). As the fundamental social unit, households organize disaster preparedness activities, linking individuals with society and communities (8–12). When facing natural disasters, households often become the most dependable support systems. Household decision-making processes significantly shape their attitudes and actions in disaster preparation (13).

Current studies on family disaster preparedness show that, despite households recognizing the necessity of implementing preventive measures, the adoption rate of such measures remains low (14–17). Individuals’ behavioral motivations and decision-making processes in response to disasters are crucial for comprehending and enhancing the effectiveness of disaster preparedness (18, 19). Existing studies have explored various psychological factors affecting household preparedness, such as risk perception (20–22), place attachment (7, 23), and self-efficacy (24, 25). Studies on the impact of psychological factors on household preparedness have reported contradictory results. For example, Ao et al. (26) found that residents’ risk perception positively affected their evacuation choice behavior. Mertens et al. (27) found that risk perception had a negative predictive effect on household tree planting and other protective behaviors. In the context of earthquakes, place attachment has been shown to strongly influence preparedness behaviors. For instance, individuals with strong ties to their homes are often less willing to evacuate, prioritizing the preservation of their residence over evacuation, as demonstrated in studies by Mishra et al. (17). Self-efficacy plays a critical role in flood preparedness, where individuals with higher self-efficacy are more likely to engage in protective measures such as installing flood barriers or developing evacuation plans (9). The impact of psychological factors on household preparedness depends on various social-economic (e.g., income, financial support), social-demographic (e.g., age, geographical location), and cultural backgrounds (26, 28–30). For example, risk perception and preparedness behaviors vary significantly across cultural and geographic contexts, such as in Eastern versus Western societies (31). Accordingly, cultural and regional differences significantly influence the psychological drivers of disaster preparedness, necessitating a comprehensive examination across diverse settings. There is a lack of systematic overview of psychological interventions, especially in terms of how psychological interventions form different preparedness attitudes under different cultural backgrounds and family characteristics. This review categorizes these psychological factors and systematically summarizes each category’s impact on household disaster preparedness. It explores how these psychological factors influence and shape household preparedness strategies, especially in diverse cultural and environmental contexts. Based on the review of research results, practical suggestions for disaster preparedness are provided from various aspects of psychological intervention. The review provides practical insights for developing effective psychological strategies to improve household preparedness and disaster coping mechanisms.

This review focuses on empirical articles about household preparedness activities for natural disasters and provides an overview of psychological factors across different cultural and disaster contexts. The remainder of the review is organized as follows: Section 2 provides an overview of the selected articles, including their socio-cultural contexts, types of disasters, and research methods and tools. Section 3 categorizes these psychological factors into four classifications based on commonalities and provides the theoretical and empirical basis for this categorization. Section 4 provides a detailed review of household preparedness under psychological interventions from the perspective of these four psychological categories. This section includes how psychological factors may promote, hinder, or not affect family preparedness and outlines the potential reasons for these varied outcomes. Section 5 summarizes the mechanisms of psychological interventions influencing family preparedness, identifies gaps in current research, and proposes directions for future studies. The review concludes with Section 6, which summarizes the review and some key points that should be considered.

## 2 Systematic review protocol

The present research uses the systematic review approach to synthesize the literature on the impact of psychological factors on household preparedness. This systematic review was conducted and reported following the standard criteria outlined in the Preferred Reporting Items for Systematic Reviews and Meta-Analyses (PRISMA) guidelines (32). The detailed process of the systematic review is elaborated upon in the following sections.

### 2.1 Search strategy

To ensure that the research included reflects the progress in psychology and disaster preparedness in recent years, the literature search for this review covers the related research published from 2017 to 2024. Three electronic databases—Web of Science, Google Scholar, and ScienceDirect—were utilized to focus on household disaster preparedness within psychological interventions. Subsequently, the search “TS = (psychological* OR psychology) AND TS = (disaster preparedness) AND TS = (household preparedness OR household emergency preparedness OR household disaster preparedness) AND TS = (natural*)” was conducted in the titles and abstracts of the articles (TS = Topic). Abstracts of conference papers, letters to the editor and data reports were not considered. In addition, we looked for 6 other pertinent publications in the 694 published articles’ references that were found during the search.

### 2.2 Inclusion and exclusion criteria

**Figure 1:**
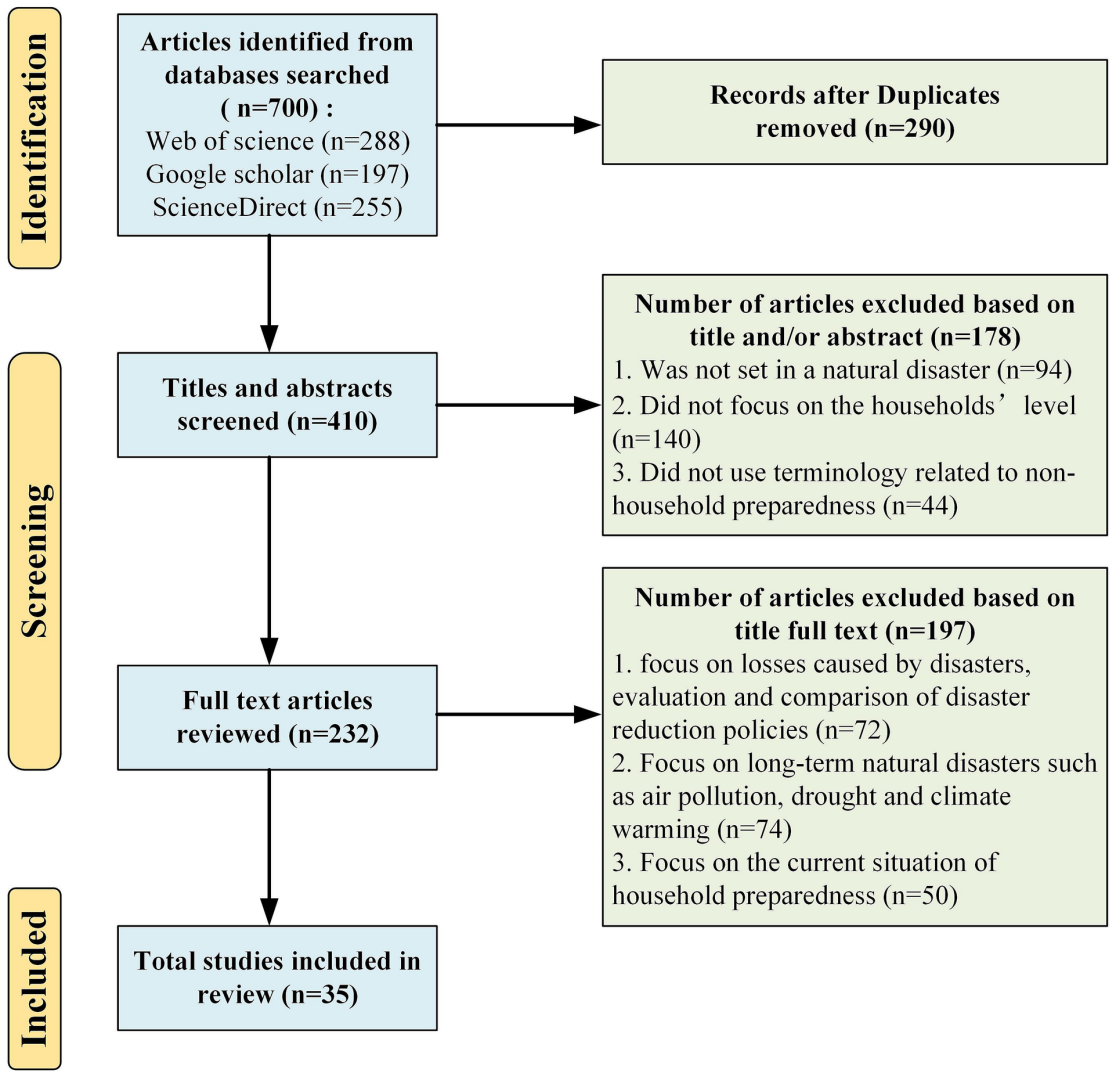
Flow chart of the decision process for selecting articles on household preparedness under psychological influence.

The following inclusion and exclusion criteria were applied to select relevant articles. The inclusion criteria were: (1) peer-reviewed papers; (2) English language; (3) full text only; (4) empirical; and (5) household preparedness for natural hazards. Articles were excluded if they: (1) utilized terminology that is not related to disaster preparedness from the household level, such as livelihood preparedness and social recovery. (2) Additionally, some articles focused on broader societal concepts, such as social vulnerability, rather than psychological factors directly related to household preparedness, were also excluded; (3) focused on long-term natural disasters such as air pollution, drought, and climate warming; (4) described the current state of household preparedness or the effectiveness of household preparedness strategies; and (5) focused on individuals’ mental health rather than the mechanism of psychological factors affecting disaster preparedness. Figure 1 demonstrates the steps in article selection, which were developed based on the PRISMA standard guidelines (33).

### 2.3 Data extraction and coding

By searching the three databases with the selected search terms, we preliminarily found 700 articles. After removing the duplicate articles, 410 articles remained, marking the first stage of refining the dataset. Next, using the exclusion criteria, 178 articles were removed based on titles and abstracts. Finally, a full-text review of the remaining 232 articles resulted in the exclusion of 197 articles, leaving 35 eligible articles for detailed analysis.

The coding scheme for the articles includes *a priori* coding and thematic coding. Based on predefined inclusion and exclusion criteria, the identified articles were coded independently by the first and the second author. Then, theory-driven thematic coding, such as the Health Belief Model (HBM) was used to categorize the psychological factors in the identified articles, establish themes, and further interpret and analyze these psychological factors. The confirmed classification is presented in section 3. The first and third author independently coded the articles into different categories. The value of Cohen’s Kappa was 0.82, indicating high agreement. Discrepancies were resolved through discussion and consensus, with arbitration by the fourth author when necessary.

### 2.4 Overview of the selected studies

**Figure 2:**
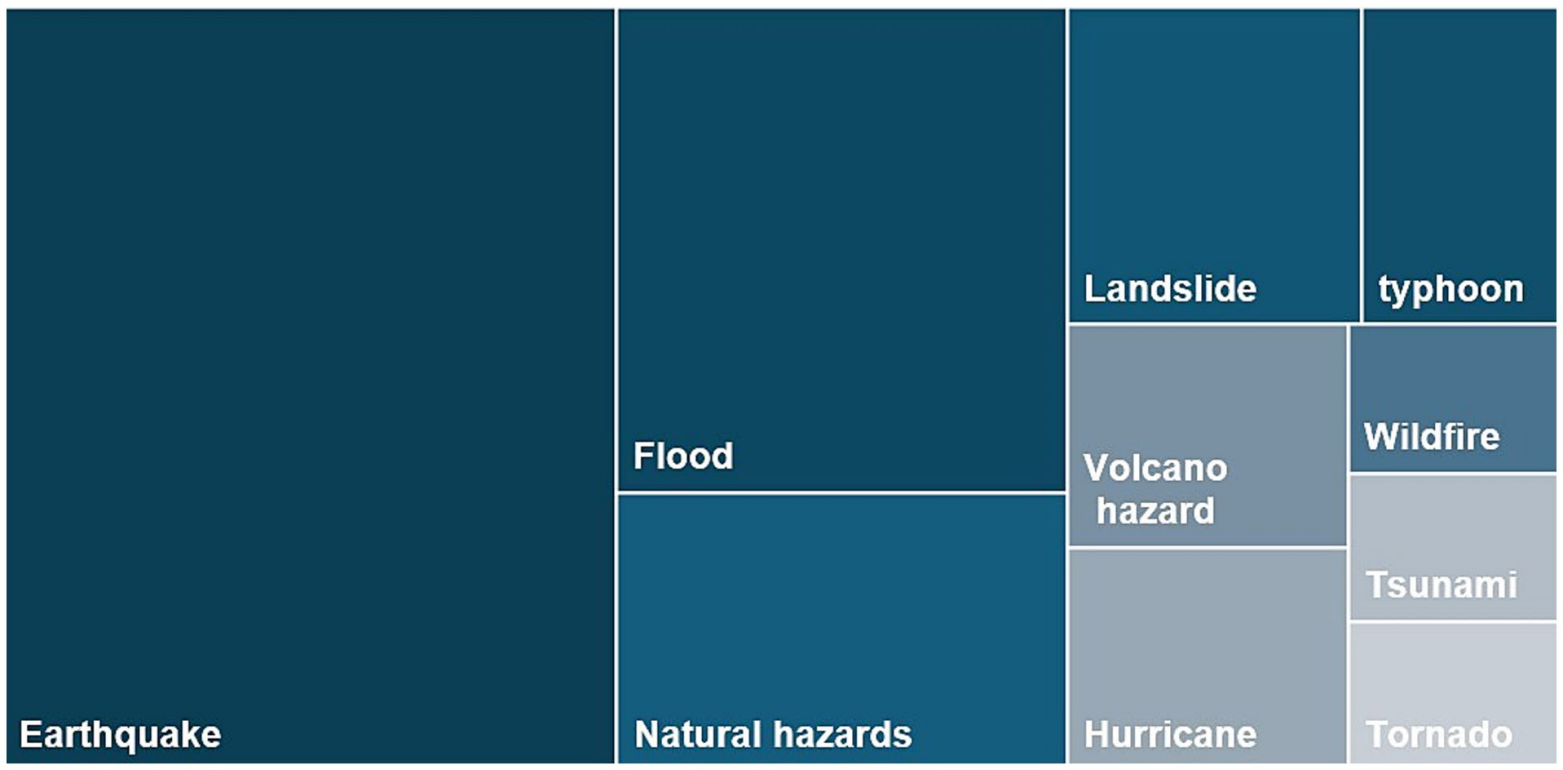
Distributions of disaster types in the reviewed studies.

**Figure 3:**
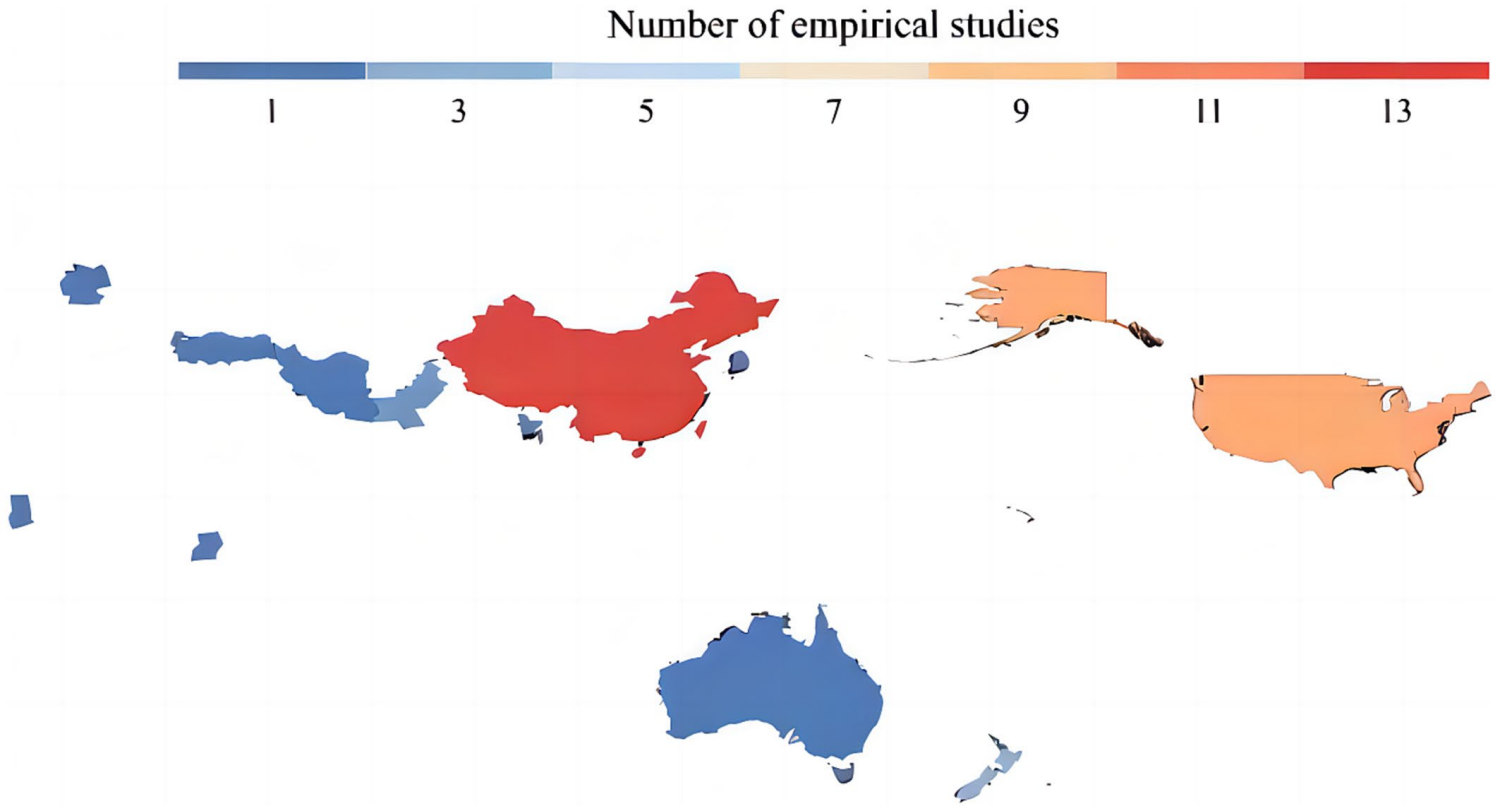
Regional distribution of the reviewed studies.

The descriptive details of the selected articles are presented in Supplementary Table 1. These details encompassed the regions and disaster types, the psychological factors examined, and the methodologies utilized in these studies. Of the selected 35 articles, general natural disasters were considered in 11% of the studies. The remaining articles focus on specific types of disasters: earthquakes (38%), floods (17%), landslides (8%), wildfires (3%), hurricanes (5%), typhoons (6%), tsunamis (3%), Tornadoes (3%), and volcanic hazards (6%) (see Figure 2). Most of the studies were conducted in China (30%) and the United States (30%), with the remainder distributed across other countries, including Korea, New Zealand, Iran, Germany, Uganda, Pakistan, Bangladesh, and Ghana (each contributing 4 to 7% of the studies) (see Figure 3).

The study designs adopted by the screened articles were categorized into cross-sectional and longitudinal designs. Among the research reviewed, only Joffe et al. (14) and Wallis et al. (34) utilized longitudinal designs, offering insightful perspectives on the dynamics of psychological factors over time. Wallis et al. (34) initially explored the link between place attachment and household preparedness by engaging participants in a place visualization task. This approach assessed whether place attachment visualization increased preparedness intentions through a follow-up survey conducted 2 weeks after the pre-intervention task. Joffe et al. (14) employed a direct intervention method to track preparedness improvements over time, facilitated through a structured “Fix-it” workshop, with follow-up assessments revealing progress at 3-and 12-month intervals. The longitudinal design facilitated the observation of sustained changes in psychological attitudes and behaviors (35). This approach offers a deeper understanding of the causality between psychological factors and preparedness, unlike the more prevalent cross-sectional studies.

Regarding methodology, questionnaires, interviews, and on-site field measurements were explored. 94% of the studies primarily employed questionnaires for data collection, with variability in sample selection and data collection methods. Specifically, the predominant approach for sample selection involved stratified random sampling. The sample selection was based on the severity of earthquake disasters (36–38), differences in economic development levels (38–40), and geographical locations (40, 95). Only Kim and Kim (23), Ghasemi et al. (25), and Wei and Lindell (41) used a nationwide population as samples. The standards for sampling were diverse, depending on the specific objectives and contextual framework.

The predominant method for questionnaire collection was face-to-face completion (3, 7, 42–45). Additionally, computer-assisted personal interviewing (CAPI) systems (46–48), online surveys (95, 45), and mail surveys (23, 49) were also utilized. Face-to-face completion and the CAPI system generally achieved higher data quality and response rates compared to other approaches. These methods required higher costs and more complex implementation. Therefore, the selection of questionnaire collection methods was based on the research’s specific requirements and the characteristics of the target sample. In addition to traditional questionnaire surveys, Becker et al. (30) further explored the reasons and influencing factors for participants implementing preparedness measures through interviews. They explored how residents interpret earthquake information. They also explained the specific process by which people prepare for earthquakes. Compared to questionnaire surveys, interviews revealed some potential factors that were previously undiscovered and considered to affect disaster preparedness.

However, it is challenging to control or standardize external variables that may affect the results when using only questionnaires or interviews. To reduce the influence of social expectations on participants when answering questions and to reflect the psychological effects more intuitively, Wallis et al. (34) and Joffe et al. (14) employed experimental interventions. Wallis et al. (34) investigated the influence of psychological factors on preparedness and the effectiveness of these interventions by comparing survey responses before and after the experiments. Joffe et al. (14) enhanced disaster preparedness willingness by increasing individuals’ attention to placing emergency supplies at home. Although these two studies primarily aimed to assess the efficacy of intervention tasks, they offered profound insights into the dynamics between psychological factors and preparedness behaviors.

The selection of a research methodology to investigate the influence of psychological factors on household preparedness depends on the study objectives. Questionnaires gather extensive data on self-reported behaviors, probing the relationship or correlation between these psychological factors and household preparedness. Behavioral observations assess and analyze participants’ actions to uncover how psychological motivation translates into behaviors. To better understand how psychological factors influence family preparedness and how psychological interventions translate into preparedness actions, it is feasible to combine questionnaire surveys and behavioral observations. The combination of research methods allows for extensive data collection and on-site verification of behaviors. Additionally, a comprehensive predictive model can be constructed by using statistical methods to analyze the relationships between the data.

## 3 Classification of psychological factors

Before delving into the detailed influence of psychological factors on household disaster preparedness, we classified them based on their mechanisms of influence. Specifically, psychological factors were identified through a thematic analysis of the reviewed studies, focusing on recurring variables such as risk perception, motivation, and place attachment. These factors were subsequently grouped into four overarching categories based on established theoretical frameworks like the Health Belief Model and Protection Motivation Theory: cognitive appraisal, motivation and intention, socio-cultural contexts, and bonds with the living environment (see Table 1). This categorization aims to group the main aspects of psychological interventions that influence household preparedness, which aims to systematically analyze how these factors, within their respective categories, independently and collectively influence household preparedness. Effective intervention strategies should be developed for different psychological mechanisms. Sections 3.1 to 3.4, respectively, elaborate on the classification of psychological factors and elucidate the rationale behind these categorizations.

**Table 1:** Classification of the psychological factors in the studies.

Categories	Psychological factors	Sources
Cognitive appraisal	Risk perception	Rosenstock (56), Rogers (52), Altarawneh et al. (47), and Kiani et al. (42)
	Hazard intrusiveness	Bodas et al. (54)
	Perceived effectiveness	Rogers (52) and Ghasemi et al. (25)
	Perceived response efficacy	Rogers (52) and Miao and Zhang (28)
Motivation and Intention	Perceived Benefits	Rostami-Moez et al. (29)
	Self-Efficacy	Zhang et al. (59) and Bandura (86)
	Trust	Zhang et al. (59) and Wei et al. (60)
Social interaction	Social norms	Berkes and Ross (92), Norris et al. (93), and Siporin (63)
	Social support	Santos et al. (66)
	Community resilience	Berkes and Ross (92) and Norris et al. (93)
Bonds with the living environment	Place attachment	Jorgensen and Stedman (68) and Ghasemi et al. (25)
	Sense of place	Hashemnezhad et al. (94) and Jorgensen and Stedman (68)

### 3.1 Classification of cognitive appraisal

Cognitive appraisal involves individuals’ subjective evaluation of disaster risks and coping resources. Four main psychological factors are classified under cognitive appraisal: risk perception, hazard intrusiveness, perceived effectiveness, and perceived response efficacy. These factors are classified based on their critical roles in modulating the perceived necessity and immediacy of actions for household disaster readiness (50). The basis for classifying these psychological factors as cognitive appraisal is as follows:

Risk perception: drawing from the Health Belief Model (HBM) (51) and Protection Motivation Theory (PMT) (52, 53), risk perception underscores an individual’s assessment of disaster threat and encompasses the appraisal of the likelihood and severity of a disaster occurring. Altarawneh et al. (47) and Kiani et al. (42) explicated risk perception as comprising both cognitive appraisals (perceived probability and perceived consequence) and affective appraisals (fear and worry), delineating it as an individual’s subjective judgment of future disaster risks.

Hazard intrusiveness: the subjective judgment of the disaster threat, highlighting the appraisal of the extent to which a disaster disrupts daily life (54). This interpretation is consistent with Griffin et al. (55) model of risk information seeking and processing. When evaluating risk information, individuals consider the weight of risk in cognition, that is, the degree of obstruction posed by the risk.

Perceived effectiveness: subjective appraisal by individuals of coping tactics. Originating from the Health Belief Model (HBM), perceived effectiveness assesses the overarching efficacy of intervention measures (56), and involves considering the possible costs, obstacles, and benefits of taking intervention measures and fostering a proactive attitude toward disaster preparedness (25).

Perceived response efficacy: subjective appraisal by individuals of specific preparedness behaviors. Originating from PMT (52), Individuals’ appraisal of the capability of a preparedness measure to mitigate disaster impacts highlights its utility in thwarting disaster consequences (28).

### 3.2 Classification of motivation and intention

Motivation and intention are the internal and external forces that drive individuals to take preparedness actions. Three main psychological factors—perceived benefits, self-efficacy, and trust—are classified under motivation and intention. These factors are classified based on their critical roles in promoting internal motivation and shaping specific behavioral intentions (57). The basis for classifying these psychological factors under motivation and intention is as follows:

**Perceived benefits:** within the HBM, perceived benefits are recognized as the principal intrinsic motivator that prompts individuals to adopt preventative measures (56). This motivator is inherently linked to an individual’s needs and desires. Subsequent research indicated that perceived benefits enhanced individuals’ recognition of the significance of implementing household disaster preparedness strategies, encouraging them to take specific actions due to the expected positive outcomes (29).

**Self-efficacy:** self-efficacy refers to an individual’s belief in their ability to successfully execute a specific task or behavior (52). According to Social Cognitive Theory (SCT), self-efficacy is a crucial internal element in regulating behaviors. A comprehensive understanding and enhancement of self-efficacy can significantly bolster an individual’s motivation to undertake specific actions (58). Moreover, Zhang et al. (59) demonstrated that self-efficacy impacts the selection of disaster preparedness activities, with family members tending to choose the preparedness measures they believe they can successfully implement.

**Trust:** in natural disaster risk scenarios, an individual’s trust in disaster preparedness measures or recommendations is an essential external motivator (24). Studies have underscored that individuals’ trust in social networks, authorities, and media directly affects their inclination toward preparedness (59, 60).

### 3.3 Classification of social interaction

Social interaction focuses on the role of social relationships and community environments in implementing household disaster preparedness behaviors. Three main psychological factors—social norms, social support, and community resilience—are classified under social interaction. These factors are classified based on their critical roles in disseminating risk information, sharing resources, and forming group cohesion (61). The basis for classifying these psychological factors under social interaction is as follows:

Social norms: specific cultural norms, or behavior rules widely accepted and followed within a community or group, are defined as social norms (62). Based on the social-ecological system theory, individual behavior is influenced by personal, community, and societal factors (63). Similarly, the theory of planned behavior (TPB) views social norms as social approval of individual behavior, representing normative beliefs about what individuals should do (64).

Social support: the essence of social support is the provision and receipt of resources through social interaction (65), comprising the network of resources and information exchange accessible to families (24, 40). Social support involves the assistance and help provided by family members, friends, neighbors, and the broader community. Social support influences family disaster preparedness through material support, informational support, and emotional reassurance (66).

Community resilience: in the domain of natural disaster preparedness, community resilience refers to the community’s capacity for adaptation, recovery, and transformation in the face of disasters (67). Through interactions within and between families, communities develop collective resilience against stresses, facilitating efficient resource distribution and crisis management, influencing families’ disaster adaptability and sustainable development capacity.

### 3.4 Classification of bonds with the living environment

Bonds with the living environment emphasize the dynamic relationship between people and their surroundings, whether in the natural environment, built environment, or cultural background. Two main psychological factors—place attachment and sense of place—are categorized under bonds with the living environment. These factors are classified based on their critical roles in motivating disaster preparedness behaviors such as environmental conservation and home maintenance activities (68, 69). The basis for classifying these psychological factors under bonds with the living environment is as follows:

Place attachment: place attachment refers to the emotional ties that a person develops with a living space (70). This emotional connection is based on long-term interactions, personal experiences and memories, and the significance of the place in people’s lives (71). In the domain of family disaster preparedness, place attachment motivates families to adopt proactive measures to protect their living environment, such as by enhancing the structural safety of their homes or increasing awareness of natural disasters (25).

Sense of place: a multidimensional construct that encapsulates an individual’s connection to their living environment and encompasses their identity identification, emotional investment, and functional dependence on their living environment (40). Place identity, place attachment, and place dependence are categorized under this construct (69, 72). Place identity and place dependence reflect an individual’s sense of identity and functional reliance on a place. Disaster preparedness strategies that consider a sense of place are more likely to be accepted by families, as these strategies acknowledge the uniqueness and functionality of the living environment (40).

## 4 Analysis of psychological factors’ intervention effects

### 4.1 Cognitive appraisal’s intervention effects

Cognitive factors are essential for comprehensively understanding natural disaster preparedness (71). This section delves into how individuals’ cognitive appraisals of natural disasters impact various preparedness measures, explicitly focusing on the impact of risk perception, hazard intrusiveness, perceived effectiveness, and perceived response efficacy on household preparedness. Studies primarily focus on earthquake-prone and flooding-prone regions, with research led by scholars from Asia, Europe, and the Americas (see Supplementary Table 1).

**Figure 4:**
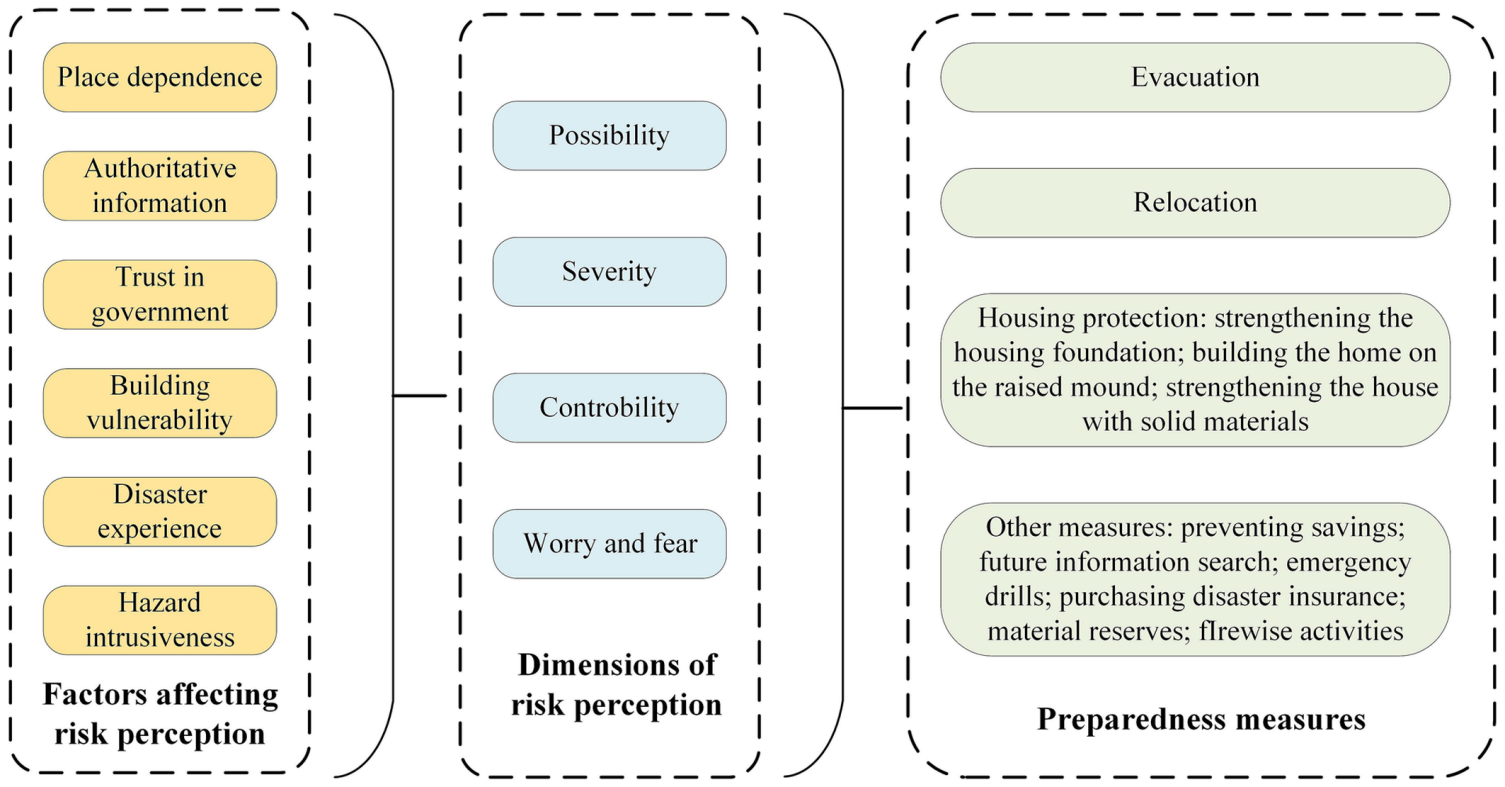
Mechanism of risk perception affecting preparedness measures.

In various natural disasters, risk perception is a crucial factor influencing disaster-affected families to take preparedness measures (73). As illustrated in Figure 4, authoritative information, disaster experience, and building vulnerability affected residents’ risk perception (73). These factors shaped individuals’ assessment of different dimensions of risk perception, such as the possibility of disaster occurrence, the severity of consequences, and emotional responses to disasters. Subsequently, these risk perceptions influenced families’ intentions to take specific preparedness measures, such as relocation and evacuation.

The impact of different dimensions of risk perception on a specific preparedness behavior was diverse and complex. For example, most studies commonly believed that perceiving the possibility of disaster occurrence and the severity of its consequences promoted the willingness to adopt protection actions and purchase insurance. In contrast, Xu et al. (22) found that residents’ assessment of the likelihood of disaster occurrence had little impact on their willingness to evacuate and relocate. The lack of effect might be due to the low frequency of earthquakes in the area where residents lived. Hazard intrusiveness (repeatedly reminding residents of the high frequency and severity of disasters) could effectively increase household preparedness by enhancing risk perception. Similarly, due to inaccurate judgments of disaster risk, disaster-affected residents’ overly high assessment of the controllability of disaster consequences has, in fact, negatively predicted the tendency to stockpile supplies and purchase disaster insurance (46). Moreover, even if residents were aware of the severe threat of disasters, they might not take structural measures (such as planting trees) or relocate (27, 69). These findings highlighted the significant impact of high place dependency and low self-efficacy on preparedness decisions.

The complex relationships outlined above suggest that disaster risk management and communication strategies need to consider specific contexts and the diversity of risk perception. Although some key factors affecting the public’s risk perception had been identified, many factors influencing the public’s assessment of the controllability of disaster consequences remained unclear (see Figure 4). Further exploration and identification of these potential influencing factors were needed. In terms of strategy implementation, overcoming psychological and socio-economic barriers to preparedness behaviors could be achieved by improving the quality of information dissemination, strengthening social support systems, and providing necessary resources and training.

Hazard intrusiveness has been found to facilitate residents’ information-seeking behavior regarding future disasters and the willingness to engage in precautionary savings and purchase disaster insurance (41, 74). Ge et al. (74) further investigated how risk exposure, specifically proximity to hazardous areas, indirectly encourages households to adopt disaster preparedness measures by increasing hazard intrusiveness. The findings indicated that disaster mitigation agencies could enhance hazard intrusiveness and increase residents’ sensitivity by regularly updating and disseminating information about disaster risk events.

Perceived response efficacy was a crucial psychological factor that promoted the willingness and behavior of households to take preventive measures (75). If individuals believe that preventative measures they can take, such as home flood-proofing, can effectively mitigate the damages caused by disasters, this belief motivates them to adopt such measures. Regarding the mechanism of perceived response efficacy, Miao and Zhang (28) found that disaster experience indirectly affects household disaster preparedness behavior by influencing perceived response efficacy. Individuals who had experienced disasters in the past 5 years might be more likely to adopt these measures due to the knowledge and confidence gained from disaster preparedness measures such as emergency plans and savings. This finding emphasized the importance of disaster experience in disaster risk education for raising public awareness and confidence in the effectiveness of disaster prevention measures.

Similarly, residents’ perceived effectiveness of Firewise activities significantly enhanced their intentions to adopt recommended actions (25). Specifically, when residents trusted that the relevant management agencies proposing Firewise activities could provide reliable information on wildfire risk mitigation, the perceived effectiveness of these measures became a significant driving force for them to take protective actions. This finding implied that authorities could effectively improve residents’ perceived effectiveness of risk mitigation measures, promoting their implementation by enhancing social trust and ensuring value alignment between disaster prevention agencies and residents.

### 4.2 Motivation and intention’s intervention effects

For households, the formation of preparedness attitudes and the execution of preparedness strategies were influenced by various motivations (57), which arise from the differences in individual characteristics and external driving forces (75). The motivations include three factors: trust, self-efficacy, and perceived benefits. Relevant studies focus on disaster types such as earthquakes, floods, hurricanes, and typhoons and were conducted by researchers from Asia and the Americas (see Supplementary Table 1). This section delves into the mechanisms through which various motivations are formed and how the motivations of family members shape their opinions and actions toward household preparedness.

Trust played a double-edged sword role in the implementation of disaster preparedness measures. Firstly, the government plays a crucial role in disaster management, particularly in Eastern cultures (76). For instance, when individuals had high trust in the government or public institutions, they were more likely to take preparatory actions based on the information and recommendations provided by these institutions (26, 60). However, excessive trust in the government could also lead to a transfer of responsibility, making households passive in disaster prevention and preparedness, as they expected the government to take charge of all preparation and response measures (30, 77). This finding suggested that the formulation of disaster risk policies needed to focus not only on the impact and mechanisms of trust in other institutions or media on household preparedness but also on exploring the factors that promote and hinder government trust across cultural backgrounds to encourage households to take appropriate self-protective measures.

High self-efficacy also encouraged households to formulate preparedness plans (28), stockpile supplies (28), learn about disaster knowledge (78), and plan evacuation behaviors (37). Individual experiences and external encouragement shaped an individual’s sense of self-efficacy. Specifically, individual experiences such as non-immediate disaster experiences enabled individuals to develop beliefs in coping with disasters, enhancing their self-efficacy (28). On the contrary, immediate disaster experiences might weaken households’ confidence in coping with disasters (48). The experience of visualizing the consequences of a disaster after a severe flash flood made residents less confident about responding to future disasters. In addition to individual characteristics and external encouragement, such as social encouragement and rewards (gift cards for group challenge winners and community praise), they have cultivated confidence in household preparedness (14, 29). These forces indirectly promoted broader participation in preparedness by increasing the social recognition of household preparedness. The above findings revealed that future research should integrate family experiences and external stimuli to promote self-efficacy. Communities and governments should pay special attention to families that have recently experienced disasters, offering external rewards through face-to-face interactions and practical activities to strengthen these families’ confidence and beliefs in coping with disasters.

Moreover, perceived benefits of self-judgment could also enhance the tendency to prepare (29). When people realized the benefits of earthquake preparedness (increased safety and reduced damage) outweighed its barriers (costs, time), they were more likely to improve their preparedness capability. The findings emphasized the importance of understanding perceived benefits, suggesting that future risk communication policies could develop more effective preparedness strategies by comparing the benefits of different intervention measures.

### 4.3 Social interaction’s intervention effects

Social interactions provided a platform for sharing information and assistance, facilitating mutual help and self-help among families (61). This section methodically explores the impact of social norms, social support, and community resilience on household preparedness. The research mainly focuses on regions prone to earthquakes and floods, such as China and Japan in Asia, the United States and Chile in the Americas, and Germany and Italy in Europe (see Supplementary Table 1). First, we examine the effects of these factors on specific disaster preparedness measures, including facilitation and hindrance. Then, we delineate the mechanisms behind these effects. Ultimately, the findings present feasible recommendations for effectively implementing disaster preparedness measures.

Social support networks provided households with information and resource mobilization channels, influencing their disaster resilience (24, 40). Different channels for obtaining social support affected families’ tendency and willingness to prepare for disasters. Social support could be formal (government or media) or informal (family or neighbors). Unlike formal support, Informal social support hindered families from preparing supplies and learning disaster response skills (24). The expectation of informal social support might reduce risk perception, thus decreasing the likelihood of families taking preventive actions. In contrast, families with a strong perception of government social support were more likely to evacuate (40). The findings underscored the importance of formal social support for family preparedness. Governments and official agencies typically provided information, issued alerts, and guided the public to prepare. On the other hand, informal support played a more significant role in the post-disaster recovery phase, providing emotional care to family members (76). Given the impact of formal support, further research could investigate other formal channels, such as the media, and their contributions to family preparedness. Comparisons could also be made between government and media support regarding specific resources provided to families for coping with disasters and minimizing losses, aiming to develop more detailed and effective preparedness measures.

Community resilience effectively predicted family preparedness awareness (23, 51). High community resilience implied that a family’s community had abundant networks and resources. Safety training provided by community organizations and a family’s geographic location could influence their perception of community resilience. Families in active community organizations and plains regions reported higher community resilience scores (51). These findings suggested that the impact of the geographical environment on community resilience should be considered in community development and planning. Appropriate land use and construction strategies should be adopted to enhance the community’s resilience. However, social norms achieved through social interaction hindered the desire of families to prepare for earthquakes (30, 79). Families typically adhered to community norms and systems, leading to information insularity and pronounced peer influence. Social norms might conflict with authorities’ recommendations (80). If a community’s consensus were to stay put or await more explicit signs of danger, individuals might be influenced to delay evacuation. This phenomenon highlighted the necessity of improving community and societal interconnectedness. Additionally, it was insufficient for communities to establish standardized guidelines emphasizing the importance of preparedness (e.g., “preparation is the best protection”). People might not be clear about the specific steps to implement these guidelines and their potential benefits.

### 4.4 Bonds with the living environment’s intervention effects

The psychological bonds between families and their living environment were manifested through place attachment and sense of place, affecting measures related to maintaining physical space, such as establishing flood barriers and reinforcing building structures (9, 25). Floods, earthquakes, and typhoons are the focus of the research, which is concentrated in high-risk disaster areas such as Japan, China, the United States, Chile, and Australia (see Supplementary Table 1). This section summarizes the mechanisms through which place attachment and sense of place influence preparedness measures (9, 25). Specifically, this section reviews the direction of influence (such as facilitation or hindrance), the specific measures affected, and the factors influencing the degree of psychological intervention.

The role of place attachment varied by the type of disaster preparedness measure. For measures aimed at protecting the home, such as reinforcing house structures and creating family protection plans, residents who had experienced wildfires and post-disaster rebuilding valued their homes and the associated memories and emotions. As a result, they were likely to implement these protective measures (25). Similarly, residents with a stronger emotional connection to their residence were more inclined to stock materials and raise disaster preparedness awareness (9). However, regarding decisions that might involve moving away from their residence, place attachment was not significantly associated with residents’ willingness to move (69). This lack of effect could be due to financial resource constraints interfering with families’ judgments on the feasibility of relocation, especially for older residents in remote areas (81, 82). Therefore, relevant agencies need to adjust subsidies based on the financial situation of the target audience when considering relocation strategies to increase their feasibility.

The impact of another psychological factor, sense of place, on household preparedness was diverse. For most preparedness measures, such as developing evacuation plans, stockpiling essentials, and reinforcing homes, sense of place is correlated with higher degrees of these preparedness activities. However, DeYoung and Peters (77) found that the sense of place was negatively correlated with these preparedness measures. The negative relationship could be due to the cultural adaptability of the scale and high population mobility. Samples with high mobility tended to have a lower sense of place, and the selected scale might not be suitable for this research sample. For specific preparedness measures such as evacuation and relocation, the impact of the sense of place is mixed. One dimension of sense of place, place dependence, was significantly negatively correlated with their willingness to relocate or evacuation (69, 72). A possible explanation was that households with a strong dependence on their place of residence due to reliance on the land for livelihood exhibited a lower willingness to relocate, even if they perceived the severity of geological disasters. In contrast, another dimension of sense of place, place identity, was positively correlated with the willingness to evacuate (72). The sense of identity that residents build with their place of residence strengthened their determination to rebuild their homes after temporary evacuation. Future policymaking, especially regarding relocation policies, could become more flexible by providing relocation compensation schemes and establishing social support systems. Additionally, integrating old cultural elements into new residential areas could help establish a new sense of place identity.

## 5 Discussion

In disaster management, household preparedness is considered a key component in reducing the impacts of natural disasters. Although people know the seriousness of the consequences of natural disasters, the motivation for household preparedness is generally insufficient. Previous studies have explored the psychological mechanisms behind insufficient or passive household preparedness. However, there needs to be a more systematic evaluation of the effectiveness of psychological interventions, particularly regarding their applicability and outcomes across different disaster contexts and family characteristics. To address this gap, a systematic review was undertaken to investigate the impact of psychological factors on household preparedness. Psychological factors are classified into four psychological intervention aspects, and practical disaster preparedness suggestions are put forward from these different perspectives.

Although there has been extensive research on the impact of psychological factors on household preparedness, the existing studies still need a thorough exploration of the psychological intervention mechanisms. For instance, one dimension of risk perception, the perceived controllability of disaster consequences, could either facilitate or hinder the implementation of preparedness measures (40, 72), but the reasons for these differences remained unknown. Future research should consider the factors influencing risk perception, particularly perceived controllability of disaster consequences. Additionally, trust was a double-edged sword in influencing residents’ preparedness actions, affected by community involvement and cultural backgrounds (83). Existing results indicated that trust in the government is generally higher in Eastern contexts (76). However, trust in different cultural and social backgrounds and its impact on family preparedness were unknown. Future studies should explore trust, especially in governments, and its varying effects on family preparedness across different cultural and social backgrounds. Lastly, although current research found that formal social support from governments could help families make reasonable evacuation decisions, informal support from friends or family negatively correlated with learning preparedness and coping skills (24, 40). The reasons for these differential outcomes were unknown. A possible explanation was that social support channels played different roles at various disaster stages. Formal social support played a crucial role in the pre-disaster warning phase, with broadcast media being a significant source of information (84). Informal social support, in contrast, often played a more crucial role in the post-disaster recovery phase (54, 85). Future research should further distinguish the roles of formal and informal social support at different disaster management stages to tailor preparedness measures effectively.

Existing research methods and tools are relatively uniform. Most studies adopted cross-sectional designs, except for Joffe et al. (14) and Wallis et al. (34). Notably, these two studies focused on exploring the effectiveness of specific preparedness strategies rather than the dynamics of psychological factors. Wallis et al. (34) utilized a longitudinal design to assess how place visualization tasks influence place attachment. Similarly, Joffe et al. (14) conducted a “Fix-it” workshop to intervene with participants directly, tracking the progress of household preparedness improvements to observe sustained changes in self-efficacy. However, the dynamic impact of psychological factors on household preparedness has not been sufficiently explored. For example, risk perception is not static; it evolves with new experiences and learning (1). However, the dynamic impact of psychological factors on household preparedness has not been sufficiently explored. For example, risk perception was not static; it evolves with new experiences and learning (1). Similarly, self-efficacy can fluctuate based on accumulated experiences and the influence of verbal persuasion (86). Adopting a longitudinal design would allow for capturing trends over time (87). Future research could benefit from such designs through long-term tracking studies and dynamic data analysis. This approach would help understand how families adapt to and respond to ongoing disaster risks and aid in assessing the effectiveness of disaster preparedness measures.

Additionally, almost all studies have used questionnaires for data collection. However, the interference of individual subjective bias, social expectations, and emotional and cognitive responses cannot be ignored (88). Recently, Yang et al. (89) combined questionnaire surveys with electroencephalogram (EEG) technology. They uniquely used EEG indicators such as voltage fluctuation and duration to reveal differences in people’s perceptions of geological disaster risks. This multimethod research design delves into risk perception from psychological and physiological dimensions, enabling researchers to explore how risk perception affects individuals’ responses to disasters and decision-making at cognitive and physiological levels. Future research could provide more comprehensive data by combining traditional questionnaires with objective physiological data, enabling more accurate analysis of the relationship between psychological factors and behaviors.

While some discussions have focused on the role of psychological factors in household preparedness behavior, a systematic framework to comprehensively consider the interaction of psychological factors with other vital elements still needs to be developed. First, this review excluded studies that primarily addressed social vulnerability. This exclusion criterion was made because social vulnerability encompasses a broader range of determinants that extend beyond psychological aspects (90, 91). However, the interaction between social and economic determinants covered by social vulnerability and psychological factors cannot be fully captured. For example, socio-economic differences can affect psychological factors such as self-efficacy and risk perception, thus indirectly affecting disaster preparedness behavior (69). Future work may need to extend and supplement specific frameworks by integrating the interactions between psychological factors and other variables, such as social and economic factors, to explore the complexity of disaster preparedness. Additionally, it should explore the weights of psychological interventions and the relationships between different psychological factors in making preparedness decisions. The lack of impact of psychological interventions on family preparedness tendencies highlights the critical role of non-psychological factors such as economic conditions and proximity to hazards. To further explore effective preparedness mechanisms and promote family preparedness, future research could consider the interactions between psychological, economic and physical factors (residential location and conditions) to develop more comprehensive and effective family preparedness strategies.

Finally, the establishment of the systematic framework requires consideration of not only diverse factors, such as non-psychological factors, but also diverse cultural backgrounds and geographical elements. It is worth noting that most of the studies reviewed were conducted in high-income countries (see Figure 3), which may restrict the applicability of the findings to low-income or disaster-prone regions. Variations in cultural norms, resource availability, and socio-economic conditions across different geographic areas can uniquely influence the psychological factors driving household preparedness. To address these challenges, future research should focus on more diverse cultural contexts, particularly in developing countries, to improve the applicability of the theoretical framework and provide more practical guidance for implementing effective disaster preparedness strategies in varying socio-economic and cultural settings.

## 6 Conclusion

This review selects the psychological factors that affect household disaster preparedness. The psychological factors involved are classified into four psychological aspects: cognitive appraisal, motivation and intention, social interaction, and bonds with the living environment. This review explores how psychological factors affect household preparedness from four psychological aspects.

Risk perception, hazard intrusiveness, perceived effectiveness, and perceived response efficacy are classified under cognitive appraisal. Perceived benefits, self-efficacy, and trust are classified as motivation and intention. Social norms, social support, and community resilience are classified as social interactions. Place attachment and sense of place are categorized as bonds with the living environment.

For cognitive appraisal, risk perception is generally a positive predictor of household preparedness, except when the low frequency of disasters renders it ineffective for household preparedness or when the sense of place among residents causes it to hinder household preparedness. Other factors, including hazard intrusiveness, perceived effectiveness, and perceived response efficacy, promote household preparedness willingness. For motivation and intention, self-efficacy and perceived benefits promote household preparedness. The influence of trust on household preparedness is mixed. The transfer of responsibility weakens the positive impact of trust in the government on household preparedness, turning its positive role into a hindrance. For social interaction, formal social support and community resilience enhance household preparedness. Informal social support and social norms hinder household preparedness. For bonds with the living environment, place attachment facilitates housing protection but hinders relocation. Sense of place enhances household preparedness measures other than permanent evacuation or relocation. The cultural adaptability of the scale and population mobility reduce the promoting effect of the sense of place.

To improve disaster preparedness, local governments and NGOs should focus on psychological interventions that increase risk perception and self-efficacy. Programs that foster community resilience and address place attachment can also be key in promoting more robust preparedness behaviors. Specifically, initiatives such as community workshops, public awareness campaigns, and training programs play a crucial role in increasing individuals’ comprehension of disaster risks and boosting their confidence in carrying out preparedness actions. Moreover, incorporating approaches that enhance community connections and address the emotional attachments to their living environments can help overcome obstacles to actions like evacuation and relocation. Given that self-reliance was identified as a significant psychological factor, disaster preparedness programs should consider cultural dimensions that emphasize self-reliance. In cultures where self-reliance is highly valued, individuals may be more proactive in taking preparedness actions. Therefore, tailoring interventions to align with cultural values of self-reliance can enhance their effectiveness. Policymakers should also consider tailoring these interventions to fit the cultural and socio-economic contexts of different regions to maximize their effectiveness.

Although every effort was made to use rigorous and comprehensive methods in conducting this review, some limitations should be considered. We focused on immediate preparedness and rapid response strategies. Therefore, we considered only non-long-term disasters. Additionally, we only reviewed articles from the Web of Science, Google Scholar, and ScienceDirect databases, which are electronic databases with high-quality scientific publications.

## Data Availability

The original contributions presented in the study are included in the article/Supplementary material, further inquiries can be directed to the corresponding author.

## References

[1] Khan SU Qureshi MI Rana IA Maqsoom A. An empirical relationship between seismic risk perception and physical vulnerability: a case study of Malakand, Pakistan. Int J Disaster Risk Reduct. (2019) 41:101317. doi: 10.1016/j.ijdrr.2019.101317

[2] Diekman ST Kearney SP O’neil ME Mack KA. Qualitative study of homeowners’ emergency preparedness: experiences, perceptions, and practices. Prehosp Disaster Med. (2007) 22:494–501. doi: 10.1017/s1049023x00005318 18709937

[3] Parida Y Agarwal GP Roy CJ Sahoo PK Nayak T. Do economic development and disaster adaptation measures reduce the impact of natural disasters? A district-level analysis, Odisha, India. Environ Dev Sustain. (2021) 23:3487–519. doi: 10.1007/s10668-020-00728-8

[4] Brown GD Largey A McMullan C. The influence of expertise on perceived and actual household disaster preparedness. Prog Disaster Sci. (2021) 9:100150. doi: 10.1016/j.pdisas.2021.100150

[5] North CS Surís AM Pollio DE. A nosological exploration of PTSD and trauma in disaster mental health and implications for the COVID-19 pandemic. Behav Sci. (2021) 11:7. doi: 10.3390/bs11010007, PMID: 33430132 PMC7827144

[6] First JM. Post-traumatic stress and depression following disaster: examining the mediating role of disaster resilience. Front Public Health. (2024) 12:1272909. doi: 10.3389/fpubh.2024.1272909, PMID: 38299076 PMC10827879

[7] Paton D. Disaster risk reduction: psychological perspectives on preparedness. Aust J Psychol. (2019) 71:327–41. doi: 10.1111/ajpy.12237

[8] Amberson T Heagele TN Wyte-Lake T Couig MP Bell SA Mammen MJ . Social support, educational, and behavioral modification interventions for improving household disaster preparedness in the general community-dwelling population: a systematic review and meta-analysis. Front Public Health. (2024) 11:1257714. doi: 10.3389/fpubh.2023.1257714, PMID: 38596429 PMC11003604

[9] Wang Z Han Z Liu L Yu S. Place attachment and household disaster preparedness: examining the mediation role of self-efficacy. Int J Environ Res Public Health. (2021) 18:5565. doi: 10.3390/ijerph18115565, PMID: 34070983 PMC8197108

[10] Rivera JD. The likelihood of having a household emergency plan: understanding factors in the US context. Nat Hazards. (2020) 104:1331–43. doi: 10.1007/s11069-020-04217-z, PMID: 32836794 PMC7407443

[11] Deschênes S Dumas C Lambert S. Household resources and individual strategies. World Dev. (2020) 135:105075. doi: 10.1016/j.worlddev.2020.105075

[12] Nurse-Clarke N Heagele T. Key factors related to household emergency preparedness among parents of newborn infants. Adv Neonatal Care. (2023) 23:229–36. doi: 10.1097/ANC.0000000000001053, PMID: 36538667

[13] Wilcox L Heagele T McNeill C. Household emergency preparedness: a multidisciplinary concept analysis. Nurs Forum. (2022) 57:305–10. doi: 10.1111/nuf.12670, PMID: 34741537

[14] Joffe H Potts HW Rossetto T Doğulu C Gul E Perez-Fuentes G. The fix-it face-to-face intervention increases multihazard household preparedness cross-culturally. Nat Hum Behav. (2019) 3:453–61. doi: 10.1038/s41562-019-0563-0, PMID: 30936428

[15] McEntire DA. Triggering agents, vulnerabilities and disaster reduction: towards a holistic paradigm. Disaster Prev Manag. (2001) 10:189–96. doi: 10.1108/09653560110395359

[16] Hoffmann R Muttarak R. Learn from the past, prepare for the future: impacts of education and experience on disaster preparedness in the Philippines and Thailand. World Dev. (2017) 96:32–51. doi: 10.1016/j.worlddev.2017.02.016

[17] Mishra S Mazumdar S Suar D. Place attachment and flood preparedness. J Environ Psychol. (2010) 30:187–97. doi: 10.1016/j.jenvp.2009.11.005

[18] Cvetković VM Ronan K Shaw R Filipović M Mano R Gačić J . Household earthquake preparedness in Serbia: a study of selected municipalities. Acta Geogr Slov. (2019) 59:27. doi: 10.3986/ags.5445, PMID: 17326625

[19] Wang C. Bracing for hurricanes: a qualitative analysis of the extent and level of preparedness among older adults. Gerontologist. (2018) 58:57–67. doi: 10.1093/geront/gnx187, PMID: 29253132

[20] Amato PR. The consequences of divorce for adults and children. J Marriage Fam. (2000) 23:5–24. doi: 10.5559/di.23.1.01

[21] Thomas TN Leander-Griffith M Harp V Cioffi JP. Influences of preparedness knowledge and beliefs on household disaster preparedness. Morb Mortal Wkly Rep. (2015) 64:965–71. doi: 10.15585/mmwr.mm6435a2, PMID: 26356729

[22] Xu D Qing C Deng X Yong Z Zhou W Ma Z. Disaster risk perception, sense of pace, evacuation willingness, and relocation willingness of rural households in earthquake-stricken areas: evidence from Sichuan Province, China. Int J Environ Res Public Health. (2020) 17:602. doi: 10.3390/ijerph17020602 31963490 PMC7013620

[23] Kim Y Kim MY. Factors affecting household disaster preparedness in South Korea. PLoS One. (2022) 17:e0275540. doi: 10.1371/journal.pone.0275540, PMID: 36194599 PMC9531828

[24] Han Z Lu X Hörhager EI Yan J. The effects of trust in government on earthquake survivors’ risk perception and preparedness in China. Nat Hazards. (2017) 86:437–52. doi: 10.1007/s11069-016-2699-9

[25] Ghasemi B Kyle GT Absher JD. An examination of the social-psychological drivers of homeowner wildfire mitigation. J Environ Psychol. (2020) 70:101442. doi: 10.1016/j.jenvp.2020.101442

[26] Ao Y Tan L Tan L Zhong J Zhang H Wang Y . Households’ earthquake disaster preparedness behavior: the role of trust in and help from stakeholders. Front Environ Sci. (2022) 10:926432. doi: 10.3389/fenvs.2022.926432

[27] Mertens K Jacobs L Maes J Poesen J Kervyn M Vranken L. Disaster risk reduction among households exposed to landslide hazard: a crucial role for self-efficacy? Land Use Policy. (2018) 75:77–91. doi: 10.1016/j.landusepol.2018.01.028

[28] Miao Q Zhang F. Drivers of household preparedness for natural hazards: the mediating role of perceived coping efficacy. Nat Hazards Rev. (2023) 24:04023010. doi: 10.1061/nhrefo.nheng-1620

[29] Rostami-Moez M Rabiee-Yeganeh M Shokouhi M Dosti-Irani A Rezapur-Shahkolai F. Earthquake preparedness of households and its predictors based on health belief model. BMC Public Health. (2020) 20:1–8. doi: 10.21203/rs.2.18198/v2 32384879 PMC7206763

[30] Becker JS Paton D Johnston DM Ronan KR. A model of household preparedness for earthquakes: how individuals make meaning of earthquake information and how this influences preparedness. Nat Hazards. (2012) 64:107–37. doi: 10.1007/s11069-012-0238-x

[31] Gierlach E Belsher BE Beutler LE. Cross-cultural differences in risk perceptions of disasters. Risk Analysis: An International Journal. (2010) *30*:1539–49. doi: 10.1111/j.1539-6924.2010.01451.x, PMID: 20626692

[32] Moher D Shamseer L Clarke M Ghersi D Liberati A Petticrew M . Preferred reporting items for systematic review and meta-analysis protocols (PRISMA-P) 2015 statement. Syst Rev. (2015) 4:1–9. doi: 10.1186/2046-4053-4-1, PMID: 25554246 PMC4320440

[33] Oláh J Krisán E Kiss A Lakne Z Popp J. PRISMA statement for reporting literature searches in systematic reviews of the bioethanol sector. Energies. (2020) 13:2323. doi: 10.3390/en13092323

[34] Wallis A Fischer R Abrahamse W. Place attachment and disaster preparedness: examining the role of place scale and preparedness type. Environ Behav. (2022) 54:670–711. doi: 10.1177/00139165211064196

[35] Van Valkengoed AM Steg L. Meta-analyses of factors motivating climate change adaptation behaviour. Nat Clim Chang. (2019) 9:158–63. doi: 10.1038/s41558-018-0371-y

[36] Xu D Zhou W Deng X Ma Z Yong Z Qin C. Information credibility, disaster risk perception and evacuation willingness of rural households in China. Nat Hazards. (2020) 17:2865–82., PMID: 31963490 10.3390/ijerph17020602 PMC7013620

[37] Buylova A Chen C Cramer LA Wang H Cox DT. Household risk perceptions and evacuation intentions in earthquake and tsunami in a Cascadia subduction zone. Int J Disaster Risk Reduct. (2020) 44:101442. doi: 10.1016/j.ijdrr.2019.101442

[38] Ge Y Yang G Wang X Dou W Lu X Mao J. Understanding risk perception from floods: a case study from China. Nat Hazards. (2021) 105:3119–40. doi: 10.1007/s11069-020-04458-y, PMID: 33424123 PMC7783707

[39] Hua C Huang SK Lindell MK Yu CH. Rural households’ perceptions and behavior expectations in response to seismic hazard in Sichuan, China. Saf Sci. (2020) 125:104622. doi: 10.1016/j.ssci.2020.104622

[40] Xu D Liu E Wang X Tang H Liu S. Rural households’ livelihood capital, risk perception, and willingness to purchase earthquake disaster insurance: evidence from southwestern China. Int J Environ Res Public Health. (2018) 15:1319. doi: 10.3390/ijerph15071319 29937510 PMC6068889

[41] Wei HL Lindell MK. Washington households’ expected responses to lahar threat from Mt. Rainier. Int J Disaster Risk Reduct. (2017) 22:77–94. doi: 10.1016/j.ijdrr.2016.10.014

[42] Kiani UB Najam FA Rana IA. The impact of risk perception on earthquake preparedness: an empirical study from Rawalakot, Pakistan. Int J Disaster Risk Reduct. (2022) 76:102989. doi: 10.1016/j.ijdrr.2022.102989, PMID: 39963269

[43] Martins VN Nigg J Louis-Charles HM Kendra JM. Household preparedness in an imminent disaster threat scenario: the case of superstorm sandy in new York City. Int J Disaster Risk Reduct. (2019) 34:316–25. doi: 10.1016/j.ijdrr.2018.11.003

[44] Mondal MS Murayama T Nishikizawa S. Examining the determinants of flood risk mitigation measures at the household level in Bangladesh. Int J Disaster Risk Reduct. (2021) 64:102492. doi: 10.1016/j.ijdrr.2021.102492

[45] Ntim-Amo G Yin Q Ankrah EK Liu Y Twumasi MA Agbenyo W . Farm households’ flood risk perception and adoption of flood disaster adaptation strategies in northern Ghana. Int J Disaster Risk Reduct. (2022) 80:103223. doi: 10.1016/j.ijdrr.2022.103223

[46] Basolo V Steinberg LJ Gant S. Hurricane threat in Florida: examining household perceptions, beliefs, and actions. Environ Hazards. (2017) 16:253–75. doi: 10.1080/17477891.2016.1277968

[47] Altarawneh L Mackee J Gajendran T. The influence of cognitive and affective risk perceptions on flood preparedness intentions: a dual-process approach. Procedia Eng. (2018) 212:1203–10. doi: 10.1016/j.proeng.2018.01.155

[48] Laudan J Zöller G Thieken AH. Flash floods versus river floods – a comparison of psychological impacts and implications for precautionary behaviour. Nat Hazards Earth Syst Sci. (2020) 20:999–1023. doi: 10.5194/nhess-20-999-2020

[49] Grover H Lindell MK Brody SD Highfield WE. Correlates of flood hazard adjustment adoption in four coastal communities. Int J Disaster Risk Reduct. (2021) 68:102728. doi: 10.1016/j.ijdrr.2021.102728, PMID: 39963269

[50] Daimon H Miyamae R Wang W. A critical review of cognitive and environmental factors of disaster preparedness: research issues and implications from the usage of “awareness (ishiki)” in Japan. Nat Hazards. (2023) 117:1213–43. doi: 10.1007/s11069-023-05909-y

[51] Sim T Han Z Guo C Lau J Yu J Su G. Disaster preparedness, perceived community resilience, and place of rural villages in Northwest China. Nat Hazards. (2021) 108:907–23. doi: 10.1007/s11069-021-04712-x

[52] Rogers RW. A protection motivation theory of fear appeals and attitude change1. J Psychol. (1975) 91:93–114. doi: 10.1080/00223980.1975.9915803, PMID: 28136248

[53] Wang Y Li C Zhang J Mao Y Li W. Protection motivation theory in predicting intentional behaviors regards schistosomiasis: a WeChat-based qualitative study. Front Public Health. (2024) 12:1295081. doi: 10.3389/fpubh.2024.1295081, PMID: 38864010 PMC11165043

[54] Bodas M Peleg K Stolero N Adini B. Risk perception of natural and human-made disasters—cross sectional study in eight countries in Europe and beyond. Front Public Health. (2022) 10:825985. doi: 10.3389/fpubh.2022.825985, PMID: 35252099 PMC8896349

[55] Griffin RJ Dunwoody S Neuwirth K. Proposed model of the relationship of risk information seeking and processing to the development of preventive behaviors. Environ Res. (1999) 80:S230–45. doi: 10.1006/enrs.1998.3940, PMID: 10092438

[56] Rosenstock IM. Historical origins of the health belief model. Health Educ Monogr. (1974) 2:328–35. doi: 10.1177/109019817400200403 299611

[57] Toyosawa J Takehashi H Shimai S. Structure of disaster preparedness motivation and its relationship with disaster preparedness behaviors. Jpn Psychol Res. (2024) 66:225–38. doi: 10.1111/jpr.12498

[58] Faryabi R Rezabeigi Davarani F Daneshi S Moran DP. Investigating the effectiveness of protection motivation theory in predicting behaviors relating to natural disasters, in the households of southern Iran. Front Public Health. (2023) 11:1201195. doi: 10.3389/fpubh.2023.1201195, PMID: 37744489 PMC10513462

[59] Zhang X Han X Dang Y Meng F Guo X Lin J. User acceptance of mobile health services from users’ perspectives: the role of self-efficacy and response-efficacy in technology acceptance. Inform Health Soc Care. (2017) 42:194–206. doi: 10.1080/17538157.2016.1200053, PMID: 27564428

[60] Wei HH Sim T Han Z. Confidence in authorities, neighborhood cohesion and natural hazards preparedness in Taiwan. Int J Disaster Risk Reduct. (2019) 40:101265. doi: 10.1016/j.ijdrr.2019.101265

[61] Hossain L Kuti M. Disaster response preparedness coordination through social networks. Disasters. (2010) 34:755–86. doi: 10.1111/j.1467-7717.2010.01168.x, PMID: 20345465

[62] Rezabeigi Davarani E Nekoei-Moghadam M Khanjani N Iranpour A Chashmyazdan M Farahmandnia H. Factors related to earthquake preparedness of households based on social-cognitive theory constructs: a systematic review. Front Public Health. (2023) 11:987418. doi: 10.3389/fpubh.2023.987418, PMID: 36875355 PMC9978524

[63] Siporin M. Ecological systems theory in social work. J Soc Soc Welf. (1980) 7:507. doi: 10.15453/0191-5096.1428

[64] Andrighetto G Vriens E. A research agenda for the study of social norm change. Philos Trans A Math Phys Eng Sci. (2022) 380:20200411. doi: 10.1098/rsta.2020.0411, PMID: 35599567 PMC9125228

[65] Ajzen I. The theory of planned behavior. Organ Behav Hum Decis Process. (1991) 50:179–211. doi: 10.1016/0749-5978(91)90020-T

[66] Santos EE Santos E Korah J Thompson JE Zhao Y Murugappan V . Modeling social resilience in communities. IEEE Trans Comput Soc Syst. (2018) 5:186–99. doi: 10.1109/TCSS.2017.2780125

[67] Shinn M Lehmann S Wong NW. Social interaction and social support. J Soc Issues. (1984) 40:55–76. doi: 10.1111/j.1540-4560.1984.tb01107.x

[68] Jorgensen BS Stedman RC. Sense of place as an attitude: lakeshore owners attitudes toward their properties. J Environ Psychol. (2001) 21:233–48. doi: 10.1006/jevp.2001.0226

[69] Paton D Johnston D. Disasters and communities: vulnerability, resilience and preparedness. Disaster Prev Manag. (2001) 10:270–7. doi: 10.1108/eum0000000005930

[70] Scannell L Gifford R. The experienced psychological benefits of place attachment. J Environ Psychol. (2017) 51:256–69. doi: 10.1016/j.jenvp.2017.04.001

[71] Ejeta LT Ardalan A Paton D Yaseri M. Emotional and cognitive factors influencing flood preparedness in Dire Dawa town, Ethiopia. Nat Hazards. (2018) 93:715–37. doi: 10.1007/s11069-018-3321-0

[72] Xu D Peng L Liu S Wang X. Influences of risk perception and sense of place on landslide disaster preparedness in southwestern China. Int J Disaster Risk Sci. (2018) 9:167–80. doi: 10.1007/s13753-018-0170-0

[73] Huang H Wang R Xiao Y Li Y Zhang Q Xiang X. Determinants of People’s secondary hazards risk perception: a case study in Wenchuan earthquake disaster areas of China. Front Earth Sci. (2022) 10:865143. doi: 10.3389/feart.2022.865143, PMID: 39963500

[74] Ge Y Peacock WG Lindell MK. Florida households’ expected responses to hurricane hazard mitigation incentives. Risk Anal. (2011) 31:1676–91. doi: 10.1111/j.1539-6924.2011.01606.x, PMID: 21449959

[75] Tohidi H Jabbari MM. The effects of motivation in education. Procedia Soc Behav Sci. (2012) 31:820–4. doi: 10.1016/j.sbspro.2011.12.148

[76] Lee DW. The expertise of public officials and collaborative disaster management. Int J Disaster Risk Reduct. (2020) 50:101711. doi: 10.1016/j.ijdrr.2020.101711

[77] DeYoung SE Peters M. My community, my preparedness: the role of sense of place, community, and confidence in government in disaster readiness. Int J Mass Emerg Disasters. (2016) 34:250–82. doi: 10.1177/028072701603400204

[78] Lee S Sadri AM Ukkusuri SV Clawson RA Seipel J. Network structure and substantive dimensions of improvised social support ties surrounding households during post-disaster recovery. Nat Hazards Rev. (2019) 20:04019008. doi: 10.1061/(asce)nh.1527-6996.0000332

[79] Thakur S Ranjitkar P Rashidi S. Modelling evacuation decisions under a threat of volcanic eruption in Auckland. Transp Res D Transp Environ. (2022) 109:103374. doi: 10.1016/j.trd.2022.103374

[80] McDonald RI Fielding KS Louis WR. Conflicting social norms and community conservation compliance. J Nat Conserv. (2014) 22:212–6. doi: 10.1016/j.jnc.2013.11.005

[81] Cretikos MA Bellomo R Hillman K Chen J Finfer S Flabouris A. Respiratory rate: the neglected vital sign. Med J Aust. (2008) 188:657–9. doi: 10.5694/j.1326-5377.2008.tb01825.x 18513176

[82] Ogrin R Robinson E Rendell K Alrababah S Fineberg D Fiddes K . “Connect local”: protocol for the evaluation of a codesigned whole of community approach to promote social connection in older adults. Front Public Health. (2024) 12:1342562. doi: 10.3389/fpubh.2024.1342562, PMID: 38846622 PMC11155451

[83] Hawkins RL Maurer K. Bonding, bridging and linking: how social capital operated in New Orleans following hurricane Katrina. Br J Soc Work. (2010) 40:1777–93. doi: 10.1093/bjsw/bcp087

[84] Ward T Beech AR. The etiology of risk: a preliminary model. Sex Abus. (2004) 16:271–84. doi: 10.1177/107906320401600402, PMID: 15560411

[85] Doust Mohammadi MM Salmani I Farahmandnia H. Social vulnerabilities among immigrants and refugees in emergencies and disasters: a systematic review. Front Public Health. (2024) 11:1235464. doi: 10.3389/fpubh.2023.1235464, PMID: 38516566 PMC10956690

[86] Bandura A. Self-efficacy: toward a unifying theory of behavioral change. Psychol Rev. (1977) 84:191–215. doi: 10.1037/0033-295x.84.2.191, PMID: 847061

[87] Linder N Rosenthal S Sörqvist P Barthel S. Internal and external factors’ influence on recycling: insights from a laboratory experiment with observed behavior. Front Psychol. (2021) 12:699410. doi: 10.3389/fpsyg.2021.699410, PMID: 34367024 PMC8340013

[88] Roopa S Rani MS. Questionnaire designing for a survey. J Indian Orthod Soc. (2012) 46:273–7. doi: 10.1177/0974909820120509s

[89] Yang M Chen Y Ma J Liu Z. The relationship between geological disaster risk perception and behavior characteristics based on electroencephalogram testing technology. Neuroquantology. (2018) 16:186–92. doi: 10.14704/nq.2018.16.5.1295

[90] Lundgren L. Jonsson A. C. Assessment of social vulnerability: a literature review of vulnerability related to climate change and natural hazards. *Environ Sociol.* (2012). Available at: https://www.semanticscholar.org/paper/Assessment-of-social-vulnerability-%3A-a-literature-Lundgren-Jonsson/5871cf2474f367fb8a2cc467af09a725c048ea53

[91] Babcicky P Seebauer S. People, not just places: expanding physical and social vulnerability indices by psychological indicators. J Flood Risk Manag. (2021) 14:e12752. doi: 10.1111/jfr3.12752

[92] Berkes F Ross H. Community resilience: toward an integrated approach. Soc Nat Resour. (2013) 26:5–20. doi: 10.1080/08941920.2012.736605

[93] Norris FH Sherrieb K Pfefferbaum B. Resilience and mental health challenges across the lifespan. Cambridge, England: Cambridge University Press (2011) 162–175.

[94] Hashemnezhad H Heidari AA Mohammad HP. Sense of place” and “place attachment. Int J Archit Urban Dev. (2013) 3:5–12. doi: 10.4324/9780429279089

[95] Han Z Wang L Cui K. Trust in stakeholders and social support: risk perception and preparedness by the Wenchuan earthquake survivors. Environ Hazards. (2020) 20:132–45. doi: 10.1080/17477891.2020.1725410

[96] Mondal MSH Murayama T Nishikizawa S. Assessing the flood risk of riverine households: a case study from the right bank of the Teesta River, Bangladesh. Int J Disaster Risk Reduct. (2020) 51:101758. doi: 10.1016/j.ijdrr.2020.101758, PMID: 39963269

[97] Li Y Greer A Wu HC. Applying the extended parallel process model to understand households’ responses to tornado and earthquake risks in Oklahoma. Risk Anal. (2024) 44:408–24. doi: 10.1111/risa.14176, PMID: 37296491

