# Supplementary material for: Psychological influences and implications for household disaster preparedness: a systematic review
Source: Front Public Health. 2025 Mar 14;13:1457406. doi: 10.3389/fpubh.2025.1457406 (PMC11949887; doi:10.3389/fpubh.2025.1457406)
Supplement: Supplementary file 1 [file Data_Sheet_1.docx]

Supplementary Material

**Psychological influences and implications for household disaster preparedness: A systematic review**

**Minrui Ni, Liang Xia*****, Xinru Wang, Yixuan Wei, Xiaofei Han, Yiqiao Liu, Song Pan**

*** Correspondence:** Liang Xia: Liang.Xia@nottingham.edu.cn

# Supplementary Figures and Tables

## Supplementary Tables

| Table 1 Descriptive details of the articles selected for systematic review | | | | |
| --- | --- | --- | --- | --- |
| **Reference information** | **Disaster types** | **Region** | **Psychological variable(s) examined** | **Methodology** |
| Xu et al. (22) | Earthquake | Three earthquake-stricken areas, China | Risk perception; place attachment | Questionnaire |
| Xu et al. (35) | Earthquake | The worst-hit areas, China | Risk perception | Questionnaire |
| Kim and Kim (23) | Natural hazards | South Korea | Community resilience | Structured online survey |
| Han et al. (24) | Earthquake | Tibetan area in China | Risk perception, social support, trust | Questionnaire |
| Han et al. (51) | Earthquake | One of the 41 severely damaged countries, China | Risk perception | A household survey |
| Xu et al. (39) | Landslide | Geological hazard-threatened areas, China | Risk perception, sense of place, social support | Questionnaire |
| Wang et al. (9) | Flood and typhoon | Shandong Province, China | Place attachment; self-efficacy | Questionnaire |
| Ghasemi et al. (25) | Wildfire | In three wildland-urban Interface areas, the United States | Risk perception; place attachment; perceived effectiveness | Questionnaire |
| Hua et al. (38) | Earthquake | Sichuan, China | Risk perception | Questionnaire |
| Khan et al. (52) | Earthquake | Pakistan | Risk perception | Semi-structured questionnaires |
| Kiani et al. (43) | Earthquake | Urban, peri-urban, and rural areas of Rawalakot. Pakistan | Risk perception | Structured questionnaires |
| Martins et al. (44) | Typhoon | New York City, the United States | Risk perception | Random digit dialing survey |
| Mondal et al. (53) | Flood | Teesta River in Bangladesh | Risk perception | Questionnaire and interview |
| Ntim-Amo et al. (46) | Flood | Northern Ghana | Risk perception | Structured questionnaire |
| Wei and Lindell (42) | Volcano hazard | Lahars, the United States | Risk perception; hazard intrusiveness | Mail survey |
| Grover et al.(50) | Flood | Four coastal communities in the United States | Risk perception | Questionnaire |
| Xu et al. (39) | landslide | Hazard-threatened areas in southwestern China. | Risk perception; sense of place | Questionnaire |
| Wei et al. (54) | Natural hazards | A natural-hazards-prone region in China | Risk perception; trust | Questionnaire |
| Miao and Zhang (28) | Natural hazards | The United States | Self-efficacy; perceived response efficacy | A national survey |
| Basolo et al. (47) | Hurricane | Florida, the United States | Risk perception | Telephone survey |
| Rostami-Moez et al. (29) | Earthquake | Hamadan province, Iran | Self-efficacy; perceived benefits | Questionnaire |
| Xu et al. (40) | Earthquake | Severe disaster counties in China | Risk perception | Cross-sectional survey |
| Merten et al. (27) | landslide | Uganda | Risk perception; self-efficacy | Cross-sectional household survey |
| Buylova et al. (36) | Earthquake and Tsunami | Cascadia Subduction Zone, the United States | Risk perception; self-efficacy | Survey |
| Thakur et al. (55) | volcanic eruption | Auckland, New Zealand | Risk perception; social norm | Online behavioral expectations questionnaire |
| Laudan et al. (49) | Flood | Germany | Risk perception; self-efficacy; perceived response efficacy | Computer-aided telephone interviews |
| Wallis et al. (33) | Natural hazards | Wellington, New Zealand | Place attachment | longitudinal intervention, 5-point Likert scale |
| Li et al. (56) | Tornado and earthquake | Oklahoma, the United States | Risk perception; self-efficacy; perceived response efficacy | Cross-sectional household survey |
| Becker et al. (30) | Earthquake | Three urban locations in New Zealand | Risk perception; self-efficacy; social norm | Qualitative interviews |
| Joffe et al. (14) | Earthquake | The United States, Türkiye | Self-efficacy | Cross-cultural, longitudinal intervention experiment and survey |
| Altarawneh et al. (48) | Flood | Southeast Queensland, Australia | Risk perception | Cross-sectional scale |
| Ge et al. (57) | Hurricane | Florida, the United States | Risk perception; hazard intrusiveness | Survey |
| Sim et al. (58) | Earthquake | Rural villages in northwest China | Community resilience | The CART (Communities Advancing Resilience Toolkit) |
| Ge et al. (37) | Flood | Urban and peri-urban areas of Nanjing, China | Risk perception; trust | Questionnaire |
| Ao et al. (26) | Earthquake | Rural villages affected by the earthquake in China | Trust | Field household surveys |
